# Does the registration system reform reduce the finance sector’s risk spillover effect in China’s stock market—Causal inference based on dual machine learning

**DOI:** 10.1371/journal.pone.0326607

**Published:** 2025-06-18

**Authors:** Yuxi Zhang, Weidong Li, Shijun Dong

**Affiliations:** 1 Beijing Jiaotong University, Beijing, China; 2 Beijing Laboratory of National Economic Security Early-warning Engineering, Beijing Jiaotong university, Beijing, China; 3 School of Finance, Southwestern university of Finance and Economics, Chengdu, China; Thammasat University, THAILAND

## Abstract

With growing uncertainty in global trade, improving access to domestic capital markets has become an important way to manage financial risk spillovers. This study examines how the registration system reform affects the finance sector's risk spillovers to other 10 sectors in China’s stock market using a dual machine learning model. The findings include: (1) The finance sector's risk spillovers vary over time and are heterogeneous. Risk spillovers rapidly intensify two months after the outbreak of the COVID-19 pandemic, with the average net ∆CoVaR value changing from −0.001 to −0.006. The finance sector mainly accepts risk from the public utility sector and exports risk to the other 9 sectors, with the highest spillovers going to the communication and information technology sectors, showing extreme net ΔCoVaR values around −0.02. (2) The registration system reform increases the finance sector's risk spillover effect, and this conclusion remains the same after a series of robustness tests. (3) Sector heterogeneity tests show that the reform boosts the finance sector's risk spillovers to cyclical sectors and sectors with a low proportion of strategic emerging companies but reduces risk spillovers to midstream and supportive sectors. Finally, some suggestions and implications are proposed.

## 1. Introduction

In recent years, China’s stock market has experienced over 20 times of the “thousand shares drop to the limit” phenomenon, which is often caused by significant declines in certain sectors (e.g. the finance sectors), combined with redemption pressures and spreading pessimism among investors, leading to a broader market downturn. Scholars have taken note of these trends, for instance, Wu (2019) highlighted the increasing infectivity within sectors in the stock market, while Zhang et al. (2020) found that systemic and inter-industry risk spillovers intensify during market crashes [1–2]. Risk contagion serves as an important warning for systemic risks (Acemoglu et al., 2015; Yang et al., 2016) [3–4]. Given the growing interconnections among sectors and the rise in investors tracking industry indices, studying risk spillover effects at the sector level helps us better understand the paths, directions, and characteristics of China’s stock market, which is key for managing systemic risks.

In the era of globalization, financial risk spillovers between countries have garnered significant attention from scholars and governments. Tian et al. (2007) and Bodart et al. (2009) found that stock markets in Europe and America influence the volatility of China’s market [5–6]. However, with the onset of the Sino-US trade war, a trend of deglobalization has emerged. The decoupling of product trade markets has led to a similar decoupling in capital markets, widening the divergence between the Shanghai Composite Index and the Dow Jones Index. For instance, from 2017 to 2023, the Shanghai Composite Index fell by 4.16%, while the Dow Jones rose by 90.72%. In this context, China’s stock market volatility is increasingly independent of European and American markets, but more on itself.

Among the sectors, the finance sector plays a key role as an intermediary between capital and the real economy. While it supports liquidity and counter-cyclical functions, it can also amplify risks during financial crises, triggering a chain reaction. For example, the 2007 US subprime mortgage crisis spread rapidly, leading to a global financial collapse. Therefore, this study focuses on the finance sector, aiming to explore the attributes of its risk spillovers and offer insights for managing risks in China’s stock market.

In 2019, China established the Sci-Tech Innovation Board (STAR) market, initiating the registration system reform, which expanded to the ChiNext market in August 2020 and to the entire stock market by February 2023. This market-oriented reform eliminates power intervention, promoting efficient capital resource allocation (Chan et al., 2004; Tian, 2011) [7–8]. It introduced key changes such as allowing loss-making companies, stocks with different rights, and VIE-structured enterprises to list; raising the daily price limit from 10% to 20% on STAR and ChiNext markets; optimizing delisting criteria; and encouraging institutional and long-term investor participation. Scholars have found that the reform improves information disclosure (Qin et al., 2021; Deng et al., 2024) [9–10], mitigates agency problems and reduces investor sentiment (Wang et al., 2025) [11], curbs post-IPO speculation (Tang et al., 2024) [12], and supports mutual fund development (Sun et al., 2022) [13]. However, the reform's requirement for minimum revenue growth and research expenditure for companies listed on STAR and ChiNext could lead to speculation and manipulation of accounting figures, compromising information quality. Overall, the reform promotes research and development, eases listing and trading, enhances information disclosure, and fosters institutional investors and value investing. But there is a lack of research on the reform's impact on the risk spillover effects in the market.

The motivation of the manuscript is to study: (1) What are the characteristics of the finance sector's risk spillover effect to other sectors in China's stock market amidst increasing uncertainty in international trade? (2) What impact has the registration system reform had on the finance sector's risk spillovers? The aim is to provide theoretical insights and empirical evidence for preventing systemic risks in China's stock market and enhancing the effectiveness of the registration system reform.

The subsequent structure is arranged as follows. The second part is the literature review. The third part is the research methodology, sample, and data. The fourth part is the empirical results. The fifth part is the heterogeneity analysis. The sixth part is conclusions and implications.

## 2. Literature review

### 2.1. The research of risk spillover effects

Many scholars, both domestic and international, have studied the risk spillover effects between countries, markets, and industries. When it comes to countries, most studies focus on the risk spillover effects between developed and developing nations. Research shows that financial markets in developed countries like the U.S., Europe, and Japan are often the source of risks, which are then transmitted to developing markets in countries like China and Southeast Asia (Xu et al., 2018; Ahrend et al., 2014; Qian et al., 2023) [14–16]. Some studies also explore risk spillovers within emerging markets. For example, Zhao et al. (2022) found that Chinese banks share two-way risk contagion with banks in East Asia, Southeast Asia, South Asia, West Asia, and Central Asia [17]. In terms of different types of markets, studies show that markets like stocks, bonds, futures, currency, and gold are interconnected. For example, Xie et al. (2023) identified stocks, funds, and futures as major risk transmitters, while bonds, gold, and shipping serve as safe havens [18]. Cui et al. (2024) also found risk spillovers between the oil, natural gas, gold, and stock markets in Palestine and Israel [19]. Some studies focus on the sector level as well. Shahzad et al. (2017) found that risk interdependence is highest in the basic materials industry and lowest in utilities, with risk spillovers peaking during the 2007–2008 financial crisis [20]. Zhang et al. (2020) observed that systemic and inter-sector risk spillovers increase during market crashes [2]. However, some studies show different results. For example, Li (2007) found no evidence of financial contagion between the stock markets of China and the United States [21].

Scholars have also studied the characteristics of risk spillover effects. They found that risk spillovers change over time and tend to increase during financial crises (Guhathakurtha et al., 2020) [22], major policy announcements or risk events (Li et al., 2021) [23], public health crises (Yang et al., 2020; Lim et al., 2024) [24–25], and other emergencies like war (Cui et al., 2024) [19]. Additionally, researchers have found that risk spillover effects are asymmetric (Xu et al., 2018; Su, 2017; Cai et al., 2017; Luo et al., 2021; Yao et al., 2024) [14,26–29].

### 2.2. The research of influencing factors of risk spillover effect

Studies also examine the factors that influence risk spillovers. The first factor is the fundamental correlation. When one country faces extreme risks, it can affect the fundamentals of another country through channels like trade, which then reflects in the stock market due to risk contagion (Cass D et al., 2004; Bekaert, 2014) [30–31]. The second factor is liquidity. A drop in liquidity in one market can cause liquidity to decrease in other markets, as asset holders like financial intermediaries adjust their behavior. This can result in a “liquidity depletion” across all markets, leading to a flight-to-liquidity (Cho et al., 2016) [32]. The third factor is globalization and the wake-up call hypothesis. Countries with more economic and financial integration are more vulnerable to financial market influences from other countries, causing risk contagion (Ahrend et al., 2014; Mendoza et al., 2010 ) [15,33]. According to the wake-up call theory, during a financial crisis, the fundamental characteristics of a country are more closely linked to domestic asset prices (Bekaert et al., 2014; Ahnert et al., 2022) [31,34]. The fourth factor is policy. Studies mainly focus on the impact of monetary policy (Ha, 2021; Hou et al., 2025) [35–36] and economic policy uncertainty (Li et al., 2017; Li et al., 2023; Hoque, 2022) [37–39]. For the stock market, Xu et al. (2017) studied the Shanghai-Hong Kong Stock Connect program and found that it increased bidirectional volatility spillovers between the Shanghai and Hong Kong stock markets, especially from Shanghai to Hong Kong [40]. Other studies have explored the impact of China’s securities margin trading policy on stock market volatility (Chang et al., 2014; Loi et al., 2021) [41–42].

In summary, scholars have extensively studied the risk spillover effects of financial risks and their impacts, offering valuable insights for our research. However, there are still some gaps: (1) Most research focuses on financial risk contagion between countries or regions, overlooking inter-sector risk spillover effects within domestic financial markets. (2) There is limited research on how regulatory policies influence risk spillover effects, especially regarding the newly implemented registration system reform in China’s stock market. (3) Few empirical studies have addressed the issue of multicollinearity between variables, which could lead to significant biases in the results.

This paper makes the following contributions to the existing literature: (1) From the perspective of research content, it examines risk spillover effects at a more detailed sector level, rather than focusing on countries or regions, providing a clearer view of the transmission path of the finance sector's risks. (2) It offers a fresh perspective on how the registration system reform in China’s stock market impacts the finance sector's risk spillovers, showing that the reform has increased these spillovers, and adds to research on its economic effects. (3) From the perspective of research methods, it introduces a dual machine learning model innovatively, using the data-driven nature of machine learning to make the research conclusions more scientific and reliable.

## 3. Research design

### 3.1. Measurement of spillover effect

Refer to the study by Adrian et al. (2016) [43], we use ∆CoVaR to measure the extent of risk spillovers between two assets, using $\Delta C{oVaR}_{q,t}^{i}$ to denote the risk spillover effect of asset j on asset i at moment t:


$$\Delta C{oVaR}_{q,t}^{i}={CoVaR}_{q,t}^{i|{VaR}_{q}^{j}}-{CoVaR}_{0.5,t}^{i|{VaR}_{0.5}^{j}}$$ (1)

When q = 0.5, it denotes the value at risk of financial asset j in an intermediate state.

We use DCC-GARCH model proposed by Engle (2002) calculate the ∆CoVaR values [44]. The estimation process is divided into two main steps, the first is to apply the GARCH model to each time series variable separately, the second is to estimate the dynamic correlation parameter by using the residuals of the first step's estimation. According to the definition of ∆CoVaR, the equation is as follows:


$$\Delta C{oVaR}_{q,t}^{i|j}=\gamma_{t}^{ji}\left({VaR}_{q,t}^{j}-{VaR}_{0.5,t}^{j}\right)$$ (2)

$$\gamma_{t}^{ji}=\rho_{t}^{ji}h_{t}^{i}/h_{t}^{j}$$ (3)

where $\rho_{t}^{ji}$ denotes the dynamic conditional correlation coefficient between the two assets at moment t. $h_{t}^{i}$ is the standard deviation of asset i estimated in the first step.

### 3.2. Regression model construction

This study examines how the registration system reform affects the finance sector's risk spillover effect in China’s stock market. Chernozhukov et al. (2018) highlighted the use of machine learning in analyzing policy effects, noting that dual machine learning can provide effective inferences with high-dimensional data under certain conditions [45]. Unlike traditional methods, dual machine learning offers unbiased results with a normal distribution. As a result, this study uses supervised dual machine learning (Chernozhukov et al., 2018; Zhang et al., 2023; Xing et al., 2023) to build the model [45–47].

First, construct a partial linear model:


$$Y_{i,t}=\alpha_{0}B_{i,t}+g(X_{i,t)}+\varepsilon_{i,t},\;E\left(\varepsilon_{i,t}|X_{i,t},B_{i,t}\right)=0$$ (4)

Where, $Y_{i,t}$ denotes the finance sector's net risk spillover to sector i in time t. $B_{i,t}$ is core explanatory variable, i.e., dummy variable of the implementation of the registration system reform. $X_{i,t}$ denotes multi-dimensional control variable, which are the covariates influencing the explanatory variable through function g. The specific form of g is unknown, and its estimated value $\hat{g}$ is obtained through machine learning models. $\varepsilon_{i,t}$ is error term with a conditional mean of 0. From equation (5), the estimated value of $\alpha_{0}$ is:


$${\hat{\alpha }}_{0}={\left(\frac{1}{n}{\sum B_{i,t}}^{2}\right)}^{-1}\frac{1}{n}\sum B_{i,t}(Y_{i,t}-{\hat{g}}_{i,t}\left(X_{i,t}\right))$$ (5)

Second, Construct the auxiliary equation for the partial linear model:


$$B_{i,t}=m\left(X_{i,t}\right)+\mu_{i,t},\;E\left(\mu_{i,t}|X_{i,t}\right)=0$$ (6)

Where, $m(X_{i,t})$ is the regression function of the core explanatory variable on the multi-dimensional control variable and its specific form is estimated by the machine learning models as well. $\mu_{i,t}$ is error term with conditional mean of 0.

The estimation procedure is as follows: Firstly, estimate $m(X_{i,t})$ using machine learning models and get the error term $\mu_{i,t}$=$B_{i,t}-m(X_{i,t})$. Secondly, estimate $\hat{g}(X_{i,t)}$ using machine learning models and change the form of equation (4) into $Y_{i,t}-g(X_{i,t)}=\alpha_{0}B_{i,t}+\varepsilon_{i,t}$. Thirdly, using $\mu_{i,t}$ as the instrumental variable for $B_{i,t}$ and get the unbiased coefficient estimation for $\alpha_{0}$:


$${\hat{\alpha }}_{0}={\left(\frac{1}{n}\sum \mu_{i,t}B_{i,t}\right)}^{-1}\frac{1}{n}\sum \mu_{i,t}(Y_{i,t}-\hat{g_{i,t}}\left(X_{i,t}\right))$$ (7)

### 3.3. Variables

(1) Explained variable: Based on the study by Wu et al. (2022) [48], we use the finance sector's net ∆CoVaR values to other sectors as the explained variable, which reflects the finance sector's net risk spillover effect. This is calculated by subtracting the ∆CoVaR values of other sectors to the finance sector from the ∆CoVaR values of the finance sector to other sectors. We select the daily closing prices of 11 first-level CSI Broad Market industry indexes and calculate their daily natural logarithmic returns. Then, we compute the finance sector's net ∆CoVaR values to the other 10 sectors separately and find the monthly average. The equations are as follows:

$$\begin{array}{c} \text{Daily\textbackslash{} net }\Delta C{oVaR}_{d}^{i|finance}=\Delta C{oVaR}_{d}^{i|finance}-\Delta C{oVaR}_{d}^{finance|i} \end{array}$$ (8)

Where: $\Delta C{oVaR}_{t}^{i|finance}$ denotes the risk spillovers from the finance sector to sector i at day d.

$\Delta C{oVaR}_{t}^{finance|i}$ denotes the risk spillovers from sector i to the finance sector at day d.


$$\begin{array}{c} \text{Monthly\textbackslash{} net }\Delta C{oVaR}_{t}^{i|finance}=\frac{\sum_{t=1}^{n}\text{Daily\textbackslash{} net }\Delta C{oVaR}_{t}^{i|finance}}{n} \end{array}$$ (9)

Where: t denotes the month.

n denotes the number of daily net ΔCoVaR in month t.

Monthly net $\Delta C{oVaR}_{t}^{i|finance}$ is $Y_{i,t}$ in equation (4).

[1] Core explanatory variable. We use a dummy variable for the implementation of the registration system reform as the core explanatory variable. It is set to 1 after the reform was implemented and 0 otherwise. Since the first batch of companies listed under the registration system began trading in July 2019 on the STAR market, we set the value to 1 starting in August 2019.
[2] Control variables. **A. Investment style**: Wahal et al. (2013) found that investment style can link stocks within the same style, while the connection between stocks of different styles is weaker [49]. We use the difference in the sector P/E ratio, P/B ratio, average market value, and average capital stock between the finance sector and other sectors to represent this variable (Xu et al., 2023; Chan et al., 2002 ) [40,50].**B. Investment sentiment**: Kumar et al. (2012) found that changes in investor sentiment affect the link between different stocks [51]. We use the difference in average stock trading turnover and turnover rate between sectors to represent this variable (Xu et al., 2023) [40]. For both the investment style and sentiment variables, we calculate the ratio of the finance sector to the other 10 sectors, using the larger value as the denominator. We then subtract the ratio from 1 to get the differences between the finance sector and the other sectors. Larger final values indicate larger differences. For sectors with minus P/E ratios, the value is 1. **C. Market state variables**: We use the 5-year and above loan interest rate, the M2 growth rate, and the logarithmic return of the Shanghai-Shenzhen 300 index (Adrian et al., 2016) [43]. **D. Market economic policy uncertainty**: We calculate the natural logarithmic value of the policy uncertainty index to represent this variable (Hoque, 2023) [39].

### 3.4. Data selection and descriptive statistics

Considering the availability, authoritative and representative of data, we use trading data from all 11 first-level sectors of the CSI Broad Market Industry Index in China’s stock market, covering the period from January 1, 2016, to September 30, 2024. The CSI Broad Market Industry Index is published by China Securities Index Co. Ltd. Table 1 shows the year-end closing prices of these 11 indexes from 2016 to September 2024. The control variables are sourced from the Wind database, and the economic policy uncertainty index is obtained from http://www.policyuncertainty.com. Descriptive statistics are provided in Table 2.

**Table 1:** The closing price of the 11 CSI indexes.

sector/date	2015-12-31	2016-12-30	2017-12-29	2018-12-28	2019-12-31	2020-12-31	2021-12-31	2022-12-30	2023-12-29	2024-09-30
real estate	8210	6681	6822	4824	5873	5125	4617	4049	2952	3183
energy	2106	2044	2070	1467	1586	1445	1924	2068	2251	2573
material	3629	3280	3480	2242	2764	3543	4551	3592	3111	3291
industry	4876	4038	3730	2467	2893	3970	4934	3834	3228	3393
optional consumption	6924	5659	5682	3691	4569	6026	5738	4673	4408	4894
primary consumption	8937	8768	11329	8857	14117	23695	21587	18440	15008	14900
medicine & health	11201	9608	10098	7432	9982	14999	13860	10927	9922	9289
finance	7730	7049	8074	6475	8541	8792	7980	6725	6379	8244
information technology	7945	5897	5780	3744	5861	6916	7425	4928	5156	5236
communication	7328	6332	6817	4500	6023	5602	6025	4652	5569	6063
public utility	3123	2520	2313	1910	1998	2019	2748	2287	2276	2677

**Table 2:** Descriptive statistics results.

	Variables	Obs	Mean	Std. Dev.	Min	Max
Explained variables	Spillover effect	1050	−0.003	0.004	−0.020	0.016
Core explanatory variable	Dummy variable	1050	0.590	0.492	0.000	1.000
Control variables	P/E ratio	1050	0.713	0.208	0.006	1.000
	P/B ratio	1050	0.560	0.217	0.008	0.883
	Market value	1050	0.867	0.093	0.360	0.946
	Capital stock	1050	0.834	0.241	0.001	0.975
	Turnover	1050	0.610	0.188	0.011	0.940
	Turnover rate	1050	0.361	0.197	0.000	0.840
	M2 growth	1050	0.097	0.017	0.062	0.140
	HS300 return	1050	0.001	0.056	−0.236	0.190
	Interest rate	1050	0.046	0.003	0.038	0.049
	EPU	1050	5.541	0.408	4.671	6.495

## 4. Empirical results

### 4.1. Analysis of the finance sector’s risk spillover characteristics in China’s stock market

(1) **Time-variation**

**Fig 1:**
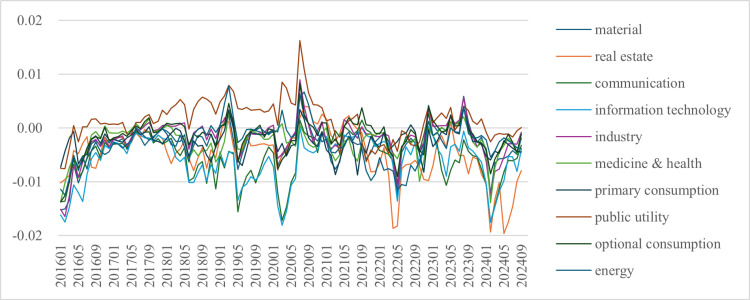
Line graph of the finance sector’s net ∆CoVaR to other sectors.

Fig 1 illustrates the changes in the finance sector's net ∆CoVaR to the other 10 sectors from January 2016 to September 2024. Overall, the risk spillovers vary and fluctuate throughout this period. These fluctuations can be roughly divided into three phases. The first phase, from January 2016 to September 2018, shows that the finance sector's net risk spillovers were high in early 2016 and gradually decreased until September 2018.

The second phase, from September 2018 to March 2021, saw an increase in risk spillover fluctuations. The ∆CoVaR values rose to near or above 0 in January 2019 before sharply dropping by May 2019. Afterward, they increased again until January 2020 and began to diverge, with sectors like public utility, energy, and medicine & health rising, while other sectors declining. The extreme values occurred around March 2020, with net ∆CoVaR values around −0.02 for communication and information technology, and around 0.01 for public utility, with other sectors falling in between. All ∆CoVaR values started rising by July 2020 and remained above 0, with public utility reaching the highest value of about 0.016. The values then dropped again until March 2021, staying between 0 and −0.01. The extreme values in 2020 were likely influenced by the COVID-19 outbreak, supporting findings that risk spillovers tend to increase during crisis periods like public health events or market crashes (Zhang et al., 2020; Lin et al., 2023) [2,52]. During this phase, the  ∆CoVaR values were the highest to the public utility sector and the lowest to the information technology and communication sectors, with other sectors in between. By the end of the period, the values began to converge.

The third phase, from March 2021 to September 2024, saw smoother fluctuations in the net ∆CoVaR values, except for the real estate, communication, and information technology sectors. These sectors experienced increased fluctuations around May 2020, February 2024, and May 2024. The real estate sector saw the lowest values, around −0.02, which is consistent with the sector's ongoing recession and deleveraging since 2020.

(2) **Sector Heterogeneity**

The spillovers also show significant sector heterogeneity (Fig 1). The most notable sector is the public utility, where net ∆CoVaR values are mostly above zero, meaning the finance sector is absorbing risks from this sector. Public utilities, which include companies providing electricity, gas, water, and heat services, are typically government-run and less reliant on the finance sector compared to other sectors. As a result, the finance sector's risk spillover to the public utility sector is somewhat limited and the finance sector's returns are influenced by the public utility sector in the stock market. On the other hand, net ∆CoVaR values to the other 9 sectors are below zero, indicating that the finance sector is exporting risks to these sectors. The net ∆CoVaR values are smallest for the communication and information technology sectors. After May 2022, the ∆CoVaR values to the real estate sector also dropped sharply and became smaller than other sectors. The communication and information technology sectors, being high-tech and asset-light, have limited risk spillovers to other sectors. The real estate sector, however, heavily relies on cash flow from the finance sector, and the increase in risk spillovers may be due to the sector's declining financial health.

### 4.2. Benchmark regression results

**Fig 2:**
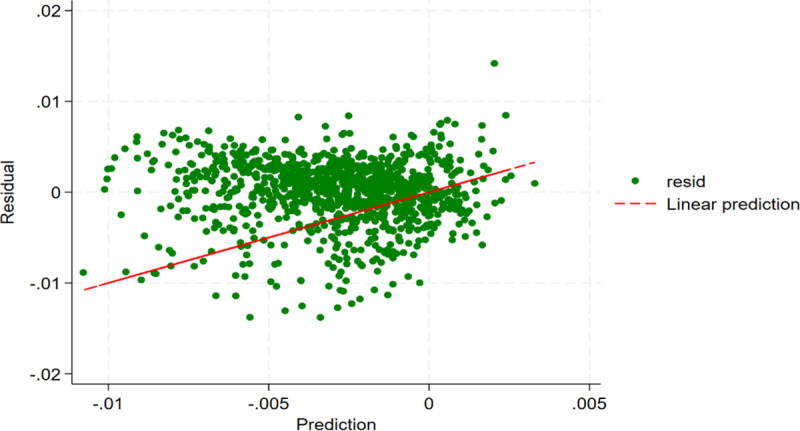
The residual plot.

We use a dual machine learning (DML) model with supervised learning to examine the impact of the registration system reform on the finance sector's risk spillovers to other sectors in China’s stock market. Before applying the DML estimation, we test for nonlinear relationships to check if the model is suitable. These tests include the RESET (Regression Specification Error Test) test and residual analysis, as shown in Table 3 and Fig 2, respectively. The null hypothesis of the RESET test is that the model is linear, but the results show p < 0.01, strongly rejecting the null hypothesis, which confirms that the model is nonlinear. The residual analysis reveals that the residual points do not appear randomly and follow a “U-shaped” pattern, further suggesting that a linear model would be biased. Therefore, a traditional linear regression model is not appropriate, and the DML model is the right choice.

**Table 3:** Test of RESET.

H0: Model has no omitted variables (the linear setting is correct)			
F (3, 1035)	6.56	p-value	0.002

The random forest algorithm and k-fold cross-validation are used for training, with time and block fixed effects included. The parameter settings are according to the system defaults, we set the number of trees to 10, min-samples-split to 2, and K to 5. The results of the dual machine learning model regressions are presented in columns (3) and (4) of Table 4. In addition, to compare the difference with the estimation results of the traditional OLS model, we give the estimation results of the OLS model at the same time, which are shown in columns (1) and (2) of Table 4. The results show that the coefficients for the reform in the OLS model are 0.0002 and −0.0333, with and without considering time fixed effects, respectively. However, these results are not statistically significant, indicating that OLS fails to capture the nonlinear relationship between the independent and dependent variables, leading to estimation bias. In contrast, the dual machine learning model shows a significantly negative coefficient for the registration system reform at the 5% significance level without considering time fixed effects (−0.0011), and at the 10% significance level with time fixed effects (−0.0006). This suggests that the reform reduces the finance sector's ∆CoVaR values to other sectors and increases the risk spillover effect, and the dual machine learning model shows better estimation results. The possible explanations for this result are as follows: The inter-sector risk spillover in the stock market is primarily driven by the fundamental and financial linkages between industries. Fundamental linkages include relationships such as supply chains and credit channels, while financial linkages are influenced by factors like cash flow and liquidity in the stock market. China’s economy has been transitioning in recent years, with a declining GDP growth rate and interest rates. The financial health of the finance sector companies has also worsened. For example, the compound debt growth rate of the finance sector companies was 7.4% from 2016 to 2019 and 10.4% from 2019 to 2023, while the compound revenue growth rate dropped from 9.65% (2016–2019) to 0.8% (2019–2023). The registration system reform improves information disclosure, raises the daily price limit, and encourages institutional investors, leading to better information transparency, more reasonable investors, and more efficient price reactions (Qin et al., 2023; Liao, 2023; Pan et al., 2023;) [9,53,54]. As a result, cash flows adjust more quickly in the stock market after the reform, making the finance sector's risks more contagious and easily transmitted to other sectors.

**Table 4:** Dual machine learning results.

Variables	OLS		DML	
(1)	(2)	(3)	(4)
Reform	0.0002	−0.0333	−0.0011**	−0.0006*
	(0.0002)	(0.0775)	(0.0005)	(0.0006)
Control	Yes	Yes	Yes	Yes
Cons	0.0083	0.0751	−0.0001	−0.0001
	(0.0075)	(0.1403)	(0.0001)	(0.0001)
Time fixed effect	No	Yes	No	Yes
Block fixed effect	Yes	Yes	Yes	Yes
N	1050	1050	1050	1050

Notes: *P < 0.1,**P < 0.05,***p < 0.01.

### 4.3. Robustness test

(1) **Reset the dual machine learning model**

To ensure the reliability of the results and minimize the impact of potential model errors, we test the robustness of our findings in two ways. First, we adjust the sample splitting ratio of the dual machine learning model from 1:4–1:2 and 1:7 to examine how the ratio might affect the conclusions, as shown in table 5, columns (1) and (2). Second, we replace the random forest algorithm with the neural network algorithms and gradient boosting to explore how the choice of machine learning algorithm might impact the results, as shown in table 5, columns (3) and (4). The test results align with the original regression findings. Specifically, the coefficients for the reform with 1/2 and 1/7 sample splits are −0.0019 and −0.0009, respectively, both significant at the 10% level. Similarly, for the neural network and gradient boosting algorithms, the coefficients are −0.0026 and −0.0006, respectively, also significant at the 10% level.

**Table 5:** Robustness test results.

Variables	Adjusting sample splitting ratio		Modifying machine learning method		Deleting abnormal observations	Considering COVID-19’s impact	Considering other parallel policy's impact
	1:2	1:7	Neural network algorithm	Gradient boosting algorithm			
	(1)	(2)	(3)	(4)	(5)	(6)	(7)
Reform	−0.0019*	−0.0009*	−0.0026*	−0.0006*	−0.0009**	−0.0018*	−0.0014***
	(0.001)	(0.0005)	(0.0013)	(0.0005)	(0.0005)	(0.0011)	(0.0004)
Control	Yes	Yes	Yes	Yes	Yes	Yes	Yes
Cons	0.0001	−0.0001	0.0011*	0.0001	−0.0001	0.0001	−0.0001
	(0.0001)	(0.0001)	(0.0006)	(0.0001)	(0.0001)	(0.0001)	(0.0001)
Time fixed effect	No	No	No	No	No	No	No
Block fixed effect	Yes	Yes	Yes	Yes	Yes	Yes	Yes
N	1050	1050	1050	1050	1050	1050	1010

Notes: *P < 0.1,**P < 0.05,***p < 0.01.

(2) **Remove outliers**

Outliers in the sample could affect the accuracy of the regression results, so we truncated all variables at the 1st and 99th percentiles, replacing values outside these ranges. We then ran the regression analysis again. The coefficient for the reform was −0.0009, significant at the 5% level, as shown in table 5, column (5). Even after removing the influence of outliers, the coefficient remained significantly negative, confirming the robustness of the original regression results.

(3) **Consider the influence of the COVID-19 epidemic**

As mentioned earlier, the COVID-19 pandemic may have influenced the finance sector's risk spillover effect. To account for this, we include a control variable for the pandemic. The COVID-19 outbreak was first reported in December 2019, and China lifted the lockdown in December 2022. Therefore, we define the impact period as December 2019 to January 2023, which is broader than the periods used by other studies. For example, Shen et al. (2023) set the impact period from January 20, 2020, to July 30, 2020 [55], while Tian et al. (2024) used December 8, 2019, to November 30, 2020 [56]. This broader period better captures the impact of the pandemic more thoroughly. The test results show that the coefficient of the reform is −0.0018 at the 10% significance level, consistent with the benchmark results, as shown in table 5, column (6). When considering the COVID-19 epidemic, the effect of the registration reform is slightly weaker, suggesting that the pandemic increased the finance sector's net risk spillover, which aligns with the “time-variation” analysis in section 4.1. To further illustrate, we compared the average risk spillovers from the finance sector to the other 10 sectors over four months (December 2019 to March 2020). The average values for these months were −0.0014, −0.0013, −0.0059, and −0.0046, with a significant drop in February 2020 compared to December 2019, indicating that the spillover effect worsened during this period.

(4) **Consider the influence of the parallel policy**

The introduction of new capital market regulations or policies could affect the inter-sector risk spillover effects in China’s stock market, potentially impacting the benchmark regression results of this study. During the test period, the Beijing Stock Exchange was established, and the CSI industry index we used includes companies listed there. To account for this, we followed Ou et al. (2024) and excluded data from July to October 2021 [57]. The results show a coefficient of −0.0014 at the 1% significance level, which matches the benchmark results, as shown in table 5, column (7). After considering the establishment of the Beijing Stock Exchange, the explanation for the registration system reform is strengthened, suggesting that the creation of the Beijing Stock Exchange helps reduce the finance sector's net risk spillovers.

## 5. Heterogeneity analysis

Based on industrial economics theory, sectors can be grouped according to their characteristics. To better understand the structural features and contagion paths of the finance sector's risk spillovers to other sectors in China’s stock market, we further categorize the 10 sectors in different ways and analyze how the registration system reform affects the finance sector's spillover impact on these various sectors.

### 5.1. Cyclical and non-cyclical sectors

We classify the 10 sectors into cyclical and non-cyclical categories based on the Shanghai-Shenzhen 300 cyclical and non-cyclical industry index. The cyclical sectors include energy, material, industry, optional consumption, and real estate, while the other belong to non-cyclical sectors. The coefficient of the reform for cyclical sectors is −0.0013 at 1% significance level without a time fixed effect, and −0.0012 at the 5% significance level with a time fixed effect, as shown in Table 6. However, for non-cyclical sectors, the coefficient is not significant. This suggests that the registration system reform has a more pronounced impact on the finance sector's risk spillover effect to cyclical sectors, increasing the risk spillover effect. Cyclical sectors are more sensitive to economic cycles than non-cyclical ones, and the finance sector's performance is more closely linked to cyclical sectors. For example, during an economic downturn, cyclical sectors are more affected by reduced demand and may need liquidity from the finance sector. However, the finance sector tends to be more cautious about lending to the real economy, potentially leading to liquidity shortages for cyclical sectors. This is reflected in stock prices, where cyclical sectors are more vulnerable, and the registration system reform amplifies this effect. Taking the energy sector as an example, from 2017 to 2019, the average growth rates of revenue and profit for energy companies were 47% and 18%, respectively, with average short-term bank loans around 44 trillion USD. In contrast, from 2020 to 2023, the growth rates dropped to 29% and 7%, with loans falling to 38 trillion USD, showing that the energy sector's financial health is worsening. The registration system's increased transparency has exposed the energy sector's debt risks, making financial institutions more cautious and reducing their willingness to lend to this sector.

**Table 6:** The influence of the reform on cyclical and non-cyclical sectors.

Variables	Cyclical sectors		Non-cyclical sectors	
	(1)	(2)	(3)	(4)
Reform	−0.0013***	−0.0012**	0.0005	0.0004
	(0.0005)	(0.0006)	(0.0005)	(0.0007)
Control	Yes	Yes	Yes	Yes
Cons	0.0001	−0.0001	−0.0001	−0.0001
	(0.0001)	(0.0001)	(0.0001)	(0.0001)
Time fixed effect	No	Yes	No	Yes
Block fixed effect	Yes	Yes	Yes	Yes
N	525	525	525	525

Notes: *P < 0.1,**P < 0.05,***p < 0.01.

### 5.2. Strategic emerging sectors and non-strategic emerging sectors

In recent years, China’s central government has identified seven “strategic emerging industries” (SEIs) that are expected to drive the country's industrial modernization and technological progress. These SEIs include next-generation information technology, biotechnology, new energy, new materials, high-end equipment, new energy vehicles, and environmental protection. We calculate the proportion of revenue and total assets from SEI companies in each sector and classify the 10 sectors into two groups: those with a high proportion of SEI companies and those with a low proportion. The high proportion group includes communication, information technology, medicine & health, industry and optional consumption, while the other belong to the low proportion group. The coefficients of the reform for the low proportion group are −0.0014 and −0.0021 at the 5% significance level, both with and without a time fixed effect, while the high proportion group is not significant, as shown in Table 7. These results suggest that the registration system reform's impact on the finance sector's risk spillover effect is more pronounced in sectors with a low proportion of strategic emerging companies, increasing the risk spillover effect. Compared to sectors with low strategic emerging companies, high strategic emerging company sectors invest more in R&D and have greater potential for internal growth, which may make them less influenced by the finance sector. For example, the 5 high proportion groups’ average R&D expense ratios from 2020 to 2023 are 3.61%, 3.79%, 4.11% and 4.41%, while the ratios for low proportion group are 0.77%, 0.81%, 0.81% and 0.85%. What’s more, the registration system reform also benefits high-tech companies by attracting investors and capital flows to these industries. As a result, sectors with fewer SEI companies are more vulnerable to the influence of the finance sector in the stock market.

**Table 7:** The influence of the reform on high strategic emerging company sectors and low strategic emerging company sectors.

Variables	High strategic emerging company proportion sectors		Low strategic emerging company proportion sectors	
	(1)	(2)	(3)	(4)
Reform	0.001	0.0009	−0.0014**	−0.0021**
	(0.0006)	(0.0006)	(0.0006)	(0.0008)
Control	Yes	Yes	Yes	Yes
Cons	−0.0001	−0.0002	0.0001	0.0001
	(0.0001)	(0.0001)	(0.0001)	(0.0001)
Time fixed effect	No	Yes	No	Yes
Block fixed effect	Yes	Yes	Yes	Yes
N	525	525	525	525

Notes: *P < 0.1,**P < 0.05,***p < 0.01.

### 5.3. Sectors at different industry chain

We classify the 10 sectors based on their position in the industry chain, following existing literature (Wu et al., 2022) [48]. Energy is in the upstream, material and industry are in the midstream, while primary consumption, optional consumption, medicine & health and public utility are downstream. Information technology, communication and real estate are supporting sectors. The reform's impact on upstream and downstream sectors is not significant. However, the coefficients for midstream sectors are 0.002 at the 5% significance level without time fixed effect and 0.0014 at the 10% significance level with time fixed effect. For supporting sectors, the coefficients are 0.0018 and 0.0019 at the 1% significance level, with and without a time fixed effect, as shown in Table 8. These results suggest that the registration system reform has a stronger effect on midstream and supporting sectors. Interestingly, the coefficients are positive, which contrasts with previous findings. This means that the reform reduces the finance sector's risk spillovers to midstream and supporting sectors. This suggests that risk spillovers in the stock market do not always follow the industry chain. Instead, the risk is more concentrated in the midstream and supporting sectors. The flow of funds, influenced by short-term speculation and macro policies targeting the development of midstream and supporting sectors, also contributes to this trend. The registration system reform has slowed the flow of funds, thus reducing risk spillovers from the finance sector to these sectors. Furthermore, the upstream sectors are more affected by external policies and resource supply, while downstream sectors are more dependent on consumer demand. As a result, they are less impacted by the finance sector compared to the midstream and supporting sectors.

**Table 8:** The influence of the reform on different sectors in the industry chain.

Variables	Upstream		Midstream		Downstream		Supportive	
	(1)	(2)	(3)	(4)	(5)	(6)	(7)	(8)
Reform	0.0007	−0.0001	0.0020**	0.0014*	0.0001	0.0003	0.0019***	0.0018***
	(0.0012)	(0.0014)	(0.0008)	(0.0007)	(0.0017)	(0.0013)	(0.0007)	(0.0006)
Control	Yes	Yes	Yes	Yes	Yes	Yes	Yes	Yes
Cons	−0.0001	−0.0002	−0.0001	−0.0002	−0.0001	−0.0001	−0.0001	−0.0001
	(0.0003)	(0.0003)	(0.0002)	(0.0002)	(0.0001)	(0.0001)	(0.0002)	(0.0001)
Time fixed effect	No	Yes	No	Yes	No	Yes	No	Yes
Block fixed effect	Yes	Yes	Yes	Yes	Yes	Yes	Yes	Yes
N	105	105	210	210	315	315	420	420

Notes: *P < 0.1,**P < 0.05,***p < 0.01.

## 6. Conclusions and implications

### 6.1. Conclusions

In this study, we analyze the trading data of all 11 first-level sectors from the CSI Broad Market Industry Index between January 1, 2016, and September 30, 2024. We calculate the daily natural logarithmic returns and use the DCC-GARCH method to calculate the finance sector's net ∆CoVaR values to the other 10 sectors, averaging these values monthly to measure the finance sector's risk spillovers. We then apply the dual machine learning method to investigate how the registration system reform affects the finance sector's risk spillovers to other sectors. The findings are as follows:

(1) The finance sector's net risk spillovers to the other 10 sectors are time varying and heterogeneous. Spillovers increased between September 2018 and March 2021, with the highest and lowest ∆CoVaR values occurring at the start of 2020, when the COVID-19 epidemic burst out. During this period, the spillovers showed clear differences, with the highest net ∆CoVaR values to the public utility sector and the lowest to the information technology and communication sectors. However, these values converged by the end of this period. After July 2020, the risk spillover effects fluctuated smoothly, except for the real estate, communication, and information technology sectors. In terms of heterogeneity, the public utility sector stands out, with the finance sector absorbing risk from it and exporting risk to the other 9 sectors. Among the 9 sectors, the finance sector's risk spillovers are most significant to communication and information technology.
(2) The registration system reform reduces the finance sector's ∆CoVaR values to other sectors while increasing the overall risk spillover effect. This may be due to the reform enhancing information transparency, encouraging more reasonable investor behavior, and improving price reactions, which make it easier for the finance sector's risks to spill over to other sectors.
(3) The heterogeneity test shows that the registration system reform has a stronger impact on the finance sector's risk spillover effect in cyclical, low strategic emerging company proportion, midstream and supportive sectors. The reform increases risk spillovers to cyclical and low strategic emerging company proportion sectors but decreases risk spillovers to midstream and supportive sectors.

### 6.2. Implications

(1) Strengthen risk prevention, management, and control in the finance sector. In recent years, China’s finance sector has faced challenges such as rising debt ratios and slowing revenue growth, leading to accumulating risks. Regulators should improve risk management and internal control systems, conduct stress tests, and simulate potential risk scenarios, including inter-sector risk spillovers. This will help identify and manage risks quickly, especially considering changes from the registration system reform, like the growing presence of emerging high-tech companies such as those in big data and artificial intelligence. Additionally, regulators could raise capital adequacy and liquidity requirements for financial companies, such as through government capital injections, to ensure they sufficient buffers to withstand market volatility and financial risks.
(2) Identify the finance sector's risk spillover paths to other sectors in the stock market, classify and process the risk spillovers according to the characteristics of different sectors. Regulators should implement a real-time risk early warning system to monitor the stock market, detect risks early, and provide warnings. At the same time, they should also develop sector-specific risk management systems, with strategies tailored to each sector's unique characteristics. The finance sector should build up its risk reserves and improve its ability to monitor and respond to risks in sectors with strong spillover effects. For example, tighter regulations and data monitoring should be applied to the real estate sector to spot potential market bubbles and credit risks early.
(3) Optimize and deepen the registration system reform. While the registration system reform improves information disclosure and price adjustment, it may also cause risks from one sector to spread more quickly to others. In the context of the registration system reform, the depth and quality of disclosures should be enhanced. Beyond financial data, listed companies should provide additional key information on business models, sector risks, market environment, governance structure, and ESG factors. This will help investors better evaluate long-term risks and sustainable growth potential, reducing excessive speculation and short-term risk spread. Additionally, investor education should be strengthened to help them better understand market volatility and financial risks, while improving their ability to identify risks and make informed investment decisions.

## Supporting information

S1 Data S1 dataset: Daily closing price and logarithmic return of the 11 CSI indexes. S2 dataset: Daily and monthly net ∆CoVaR. S3 dataset: Panel data for DML.
(ZIP)
